# Stress Cardiomyopathy in Association with Severe Agitation in Dementia

**DOI:** 10.7759/cureus.4531

**Published:** 2019-04-23

**Authors:** Hina Amin, Zachary Shepherd

**Affiliations:** 1 Internal Medicine, State University of New York Upstate Medical University, Syracuse, USA

**Keywords:** takotsubo cardiomyopathy, stress cardiomyopathy, dementia

## Abstract

Stress cardiomyopathy is a reversible left ventricular systolic dysfunction in the setting of intense emotional and physical trauma. It has rarely been described in association with the agitated state of dementia. We describe the case of a 78-year-old female with dementia who was diagnosed with stress cardiomyopathy in the setting of worsening agitation. This case underscores the importance of recognizing the non-specific manifestations of stress cardiomyopathy in this subset of patient population.

## Introduction

Takotsubo or stress cardiomyopathy (CMP) is a reversible systolic dysfunction of the left ventricle (LV) that occurs in the absence of obstructive coronary artery disease or acute plaque change within the coronary vasculature. It has been postulated to result from catecholamine excess leading to transient coronary and/or microvascular spasm and myocardial stunning. Chronic psychiatric illnesses including depression, anxiety, and substance abuse have been implicated as risk factors for this type of myocardial dysfunction. Intense physical and emotional trauma, certain acute medical conditions, and acute neurologic states are common triggers of this syndrome. However, stress CMP has also been rarely reported in the absence of extraneous triggers in elderly patients with neurodegenerative disease undergoing an exacerbation.

## Case presentation

**Figure 1:**
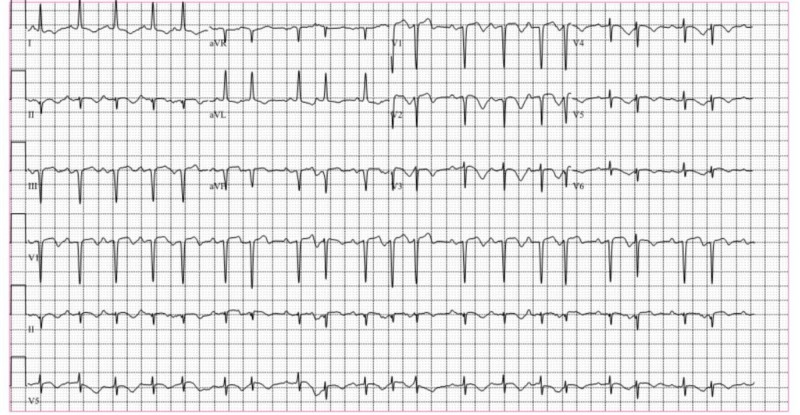
12-lead electrocardiogram (EKG) showing sinus tachycardia with a rate of 110 bpm, frequent premature atrial complexes, diffuse T wave inversions in the antero-lateral and inferior leads and poor R wave progression.

A 78-year-old female with a longstanding history of vascular dementia with Montreal Cognitive Assessment (MOCA) score of 18 and no known coronary artery disease was brought to our hospital with a chief complaint of worsening agitation, memory loss and behavioral disturbance. She was also witnessed to have paranoid delusions and sleep disturbances characterized by difficulty falling and staying asleep. This subacute decline was not precipitated by any specific emotional stressor or physical trauma. When she was brought to the hospital, the patient was alert, oriented and verbal, but combative and agitated. Her initial set of labs was unremarkable. She was given haloperidol for sedation and later switched to ziprasidone. On the second day of hospitalization, her mental status deteriorated and she appeared lethargic and disoriented while remaining hemodynamically stable and otherwise free of cardiac symptoms. Physical exam did not reveal muscle rigidity, spasticity or hyperreflexia. An electrocardiogram (EKG) was obtained which showed sinus tachycardia with a rate of 110 beats per minute, frequent premature atrial complexes, diffuse T wave inversions in the anterolateral and inferior leads, and poor R wave progression (Figure 1).

**Figure 2:**
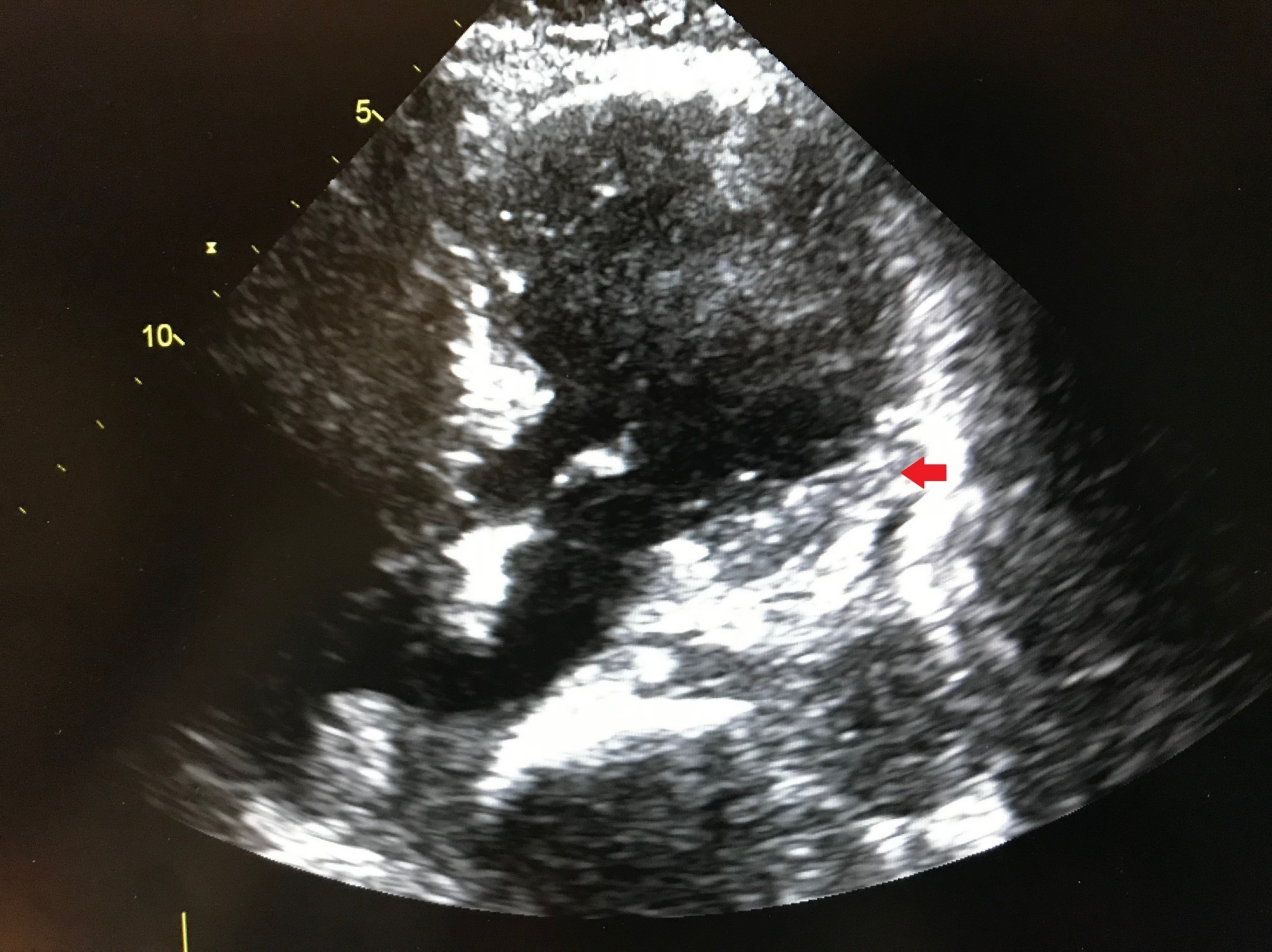
Echocardiogram (apical 2 chamber view) showing systole with apical ballooning of the left ventricle (arrow).

Troponin T was elevated to 0.09 ng/ml (reference: <0.01 ng/ml). Creatine kinase (CK) was 88 U/L (reference: 22-198 U/L), while creatine kinase-muscle/brain (CK-MB) was 2.25 IU/L (reference: 5-25 IU/L). An echocardiogram was performed which showed left ventricle ejection fraction of 20-25% and severe apical hypokinesis consistent with apical ballooning (Figure 2).

**Figure 3:**
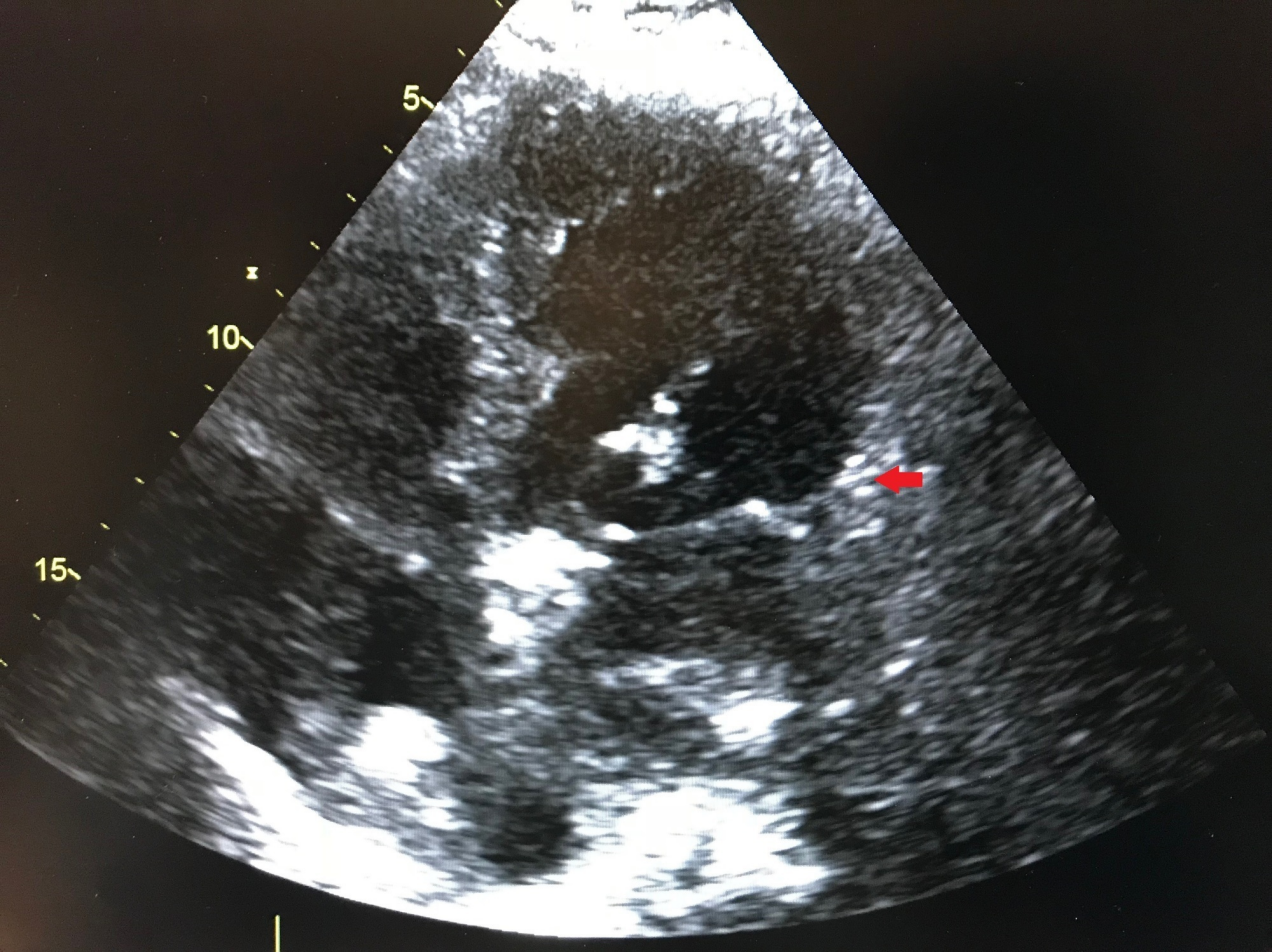
Repeat echocardiogram (apical 2 chamber view) showing mild residual hypokinesis of the left ventricle apex (arrow).

Chest X-ray showed mild to moderate pulmonary edema. Cardiac catheterization was deferred in context of severe neurodegenerative disease and we initiated a diuretic for fluid overload later followed by a beta blocker in low dose. Serial EKG demonstrated resolution of poor R wave progression and restoration of normal T wave morphology. Troponin T trended back to normal. Echocardiogram a week later showed spontaneous improvement in the left ventricle ejection fraction to 50% and minimal residual hypokinesis of the apex (Figure 3).

Chest X-ray demonstrated resolution of pulmonary edema. The patient improved clinically during this time. She continued to remain hemodynamically stable, largely free of symptoms and was discharged after receiving treatment for co-morbid conditions.

## Discussion

Stress cardiomyopathy (CMP) in patients with dementia suffering from neuropsychiatric symptoms is a rare phenomenon. The precise mechanism behind this association is not well defined; however, a catecholamine surge caused by the agitated state is a plausible explanation. Our literature search of PubMed indexed cases has yielded fewer than five reported cases. Zuin et al. reported the syndrome in an 89-year-old female with dementia with visual hallucinations in the absence of any identifiable stressors [1]. Yanagawa et al. reported stress cardiomyopathy in an 85-year-old female with dementia and Wernicke’s encephalopathy [2]. Noguchi and Yamaga reported stress cardiomyopathy in 61-year-old female with Lewy body dementia presenting with depression and apathy [3]. Cerri et al. showed transient left ventricle dysfunction in a 65-year-old female with dementia undergoing a bladder catheterization [4]. In the aforementioned case, we described the occurrence of this syndrome in a post-menopausal female with dementia and escalating agitation with psychotic features, in the absence of any extraneous emotional or physical triggers.

Our case was challenging because of the patient's advanced dementia. We were substantially limited in our ability to perform a coronary angiogram; however, spontaneous recovery of LV systolic function leads to the diagnosis. The ACC/AHA (American College of Cardiology/American Heart Association) 2018 guidelines on Takotsubo cardiomyopathy advocate the use of InterTAK diagnostic score to guide decision making. A score of 70 or higher indicates a high likelihood of Takotsubo and a transthoracic echocardiogram should be pursued in such cases [5]. Ultimately, the condition is transient and largely reversible with supportive management. The use of beta blockers is reasonable to prevent further sympathomimetic effects on the myocardium, but randomized trials supporting this hypothesis are currently lacking [5].

## Conclusions

We conclude that patients with dementia and agitation are predisposed to stress-induced CMP. It is possible that diagnosis is missed in a large subset of elderly population with dementia due to non-specific symptoms and under reporting of various cardiac symptoms in patients with advanced dementia. Clinicians must be mindful of this association when catering to elderly patients with dementia, as any acute decline can frequently be misinterpreted as worsening dementia. Further studies are needed to determine the true incidence of stress-induced CMP in this patient population and the attributable risk burden of agitation and dementia in stress-induced CMP.
